# Sleep Duration and Body Mass Index Among Adolescents: Variation by Age and Race/Ethnicity

**DOI:** 10.3390/clockssleep8030045

**Published:** 2026-07-22

**Authors:** Astrid N. Zamora, Zachary P. Gersten, Erica C. Jansen

**Affiliations:** 1 Department of Epidemiology and Population Health, School of Medicine, Stanford University, Stanford, CA 94304, USA; astridz@stanford.edu; 2 Department of Nutrition, University of California at Davis, Davis, CA 95616, USA; 3 Department of Nutritional Sciences, School of Public Health, University of Michigan, Ann Arbor, MI 48109, USA; 4 Department of Neurology, Division of Sleep Medicine, Michigan Medicine, Ann Arbor, MI 48109, USA

**Keywords:** adolescent health, body mass index, obesity, race/ethnicity, sleep health

## Abstract

Sleep characteristics have been associated with body mass index (BMI) among adolescents, but population-representative evidence on sleep timing and variation across demographic subgroups remains limited. We conducted a cross-sectional analysis of 2017–2018 California Health Interview Survey data from 880 adolescents aged 12–17 years. Weeknight sleep duration was obtained from the CHIS-derived sleep-duration variable, based on reported school-night bedtimes and school-day wake times. Sleep-timing measures included bedtime, wake time, and the derived sleep midpoint. Pooled survey-weighted linear and logistic regression models used 160 jackknife replicate weights and adjusted for age, sex, race/ethnicity, household poverty category, and survey year; sensitivity models additionally adjusted for dietary and behavioral factors. Sleep duration was not associated with BMI overall (*p* = 0.161) or with the odds of having overweight or obesity (*p* = 0.36). The association between sleep duration and BMI varied by age group (*p* for interaction = 0.042). Each additional hour of sleep was associated with a lower BMI among adolescents aged 14–17 years (β = −0.895 kg/m^2^; 95% CI: −1.464 to −0.326; *p* = 0.002), but not among those aged 12–13 years. However, there was no evidence of an interaction when age was modeled continuously (*p* for interaction = 0.36). Bedtime, wake time, and sleep midpoint were not independently associated with BMI. Exploratory evidence that the association between sleep duration and overweight or obesity varied by race/ethnicity was attenuated after adjustment for dietary and behavioral factors (*p* for interaction changed from 0.04 to 0.05). In this cross-sectional study, neither sleep duration nor sleep timing was associated with BMI in the overall sample, and sleep duration was not associated with BMI-defined weight status. Subgroup findings were not consistent across model specifications and should be examined in longitudinal studies using objective and repeated measures of sleep.

## 1. Introduction

Short sleep duration during adolescence has been consistently linked to higher adiposity and an increased risk of obesity, with particular concern that sleep loss during this developmental window may contribute to cardiometabolic vulnerability later in life [1,2,3]. Mechanistically, insufficient or mistimed sleep may influence adiposity through altered appetite-regulating hormones, impaired insulin sensitivity, increased reward-related eating, and reduced opportunity for physical activity [4]. Longitudinal evidence suggests that sleep may be particularly relevant to weight-gain trajectories during mid-to-late adolescence. For example, in a cohort of ninth-grade students followed from ages 14 to 18 years in suburban Philadelphia (U.S.) high schools, greater sleep duration was associated with lower body mass index (BMI) across the BMI distribution, with stronger associations at higher BMI percentiles [5].

Although sleep duration has been the predominant exposure examined in pediatric obesity research [4], sleep-timing measures, including bedtime, wake time, and sleep midpoint, are increasingly recognized as potential contributors through circadian mechanisms [6]. However, evidence regarding sleep timing remains mixed [7]. In a large U.S. pediatric clinical dataset drawn from well-child visits, later sleep midpoint during adolescence was associated with higher odds of overweight or obesity, whereas shorter sleep duration appeared more important for overweight and obesity in childhood [7]. These developmental differences are biologically plausible because puberty is accompanied by a normative circadian phase delay and increasing social and academic demands that may constrain sleep opportunity [7,8].

Sleep–adiposity relationships may also vary across racial and ethnic groups [9]. Structural determinants of sleep opportunity, including neighborhood conditions, household routines, and work schedules, can differ across populations, and they may modify sleep–health associations [1,9]. Most evidence on sleep and adolescent adiposity derives from national or regional samples that may not fully capture the demographic and socioeconomic heterogeneity of large U.S. states [1,9,10,11,12]. California is home to the largest population of children and adolescents in the U.S. and is characterized by substantial racial, ethnic, and socioeconomic diversity [13].

The California Health Interview Survey (CHIS) provides a unique opportunity to address this gap. CHIS is the nation’s largest state health survey and is designed to produce representative estimates for California’s population and its major racial and ethnic groups [14]. Importantly, the 2017–2018 survey cycles are the only CHIS waves to have collected detailed adolescent sleep measures, making them the most recent statewide population-representative data available for examining adolescent sleep patterns in California.

This evidence is particularly relevant given recent policy developments. In 2022, California implemented statewide school start time requirements mandating an 8:00 a.m. or later start time for public middle schools and an 8:30 a.m. or later start time for public high schools [15], consistent with recommendations from the American Academy of Pediatrics [16]. Although these policies apply only to public schools, and the CHIS data analyzed here were collected prior to policy implementation, population-level evidence on adolescent sleep patterns remains important for informing ongoing discussions about adolescent sleep health and school scheduling.

To address the lack of state-representative evidence on adolescent sleep and BMI in California, we analyzed pooled 2017–2018 CHIS adolescent data to (1) describe weeknight sleep duration and sleep timing among adolescents aged 12–17 years; (2) examine cross-sectional associations between sleep measures and BMI; (3) test whether associations differed by age group, sex, and race/ethnicity; and (4) conduct a secondary exploratory analysis of sleep duration in relation to overweight or obesity.

## 2. Results

### 2.1. Sample Characteristics

The analytic sample included 880 adolescents aged 12–17 years, representing approximately 3.14 million California adolescents. The mean (SE) age was 14.51 (0.05) years, and 48.88% were female (Table 1). Mean BMI was 22.55 (0.27) kg/m^2^; 15.47% had overweight, 17.10% had obesity (together, 32.57% had overweight or obesity). Mean weeknight sleep duration was 8.30 (0.07) hours per night. Mean sleep midpoint was 2.18 (0.06) hours after midnight (approximately 2:11 a.m.), mean wake time was 6.33 (0.06) hours after midnight (6:20 a.m.), and mean bedtime was 22.03 (0.07) hours on the linearized scale (10:02 p.m.).

### 2.2. Sleep Duration by Participant Characteristics

Adolescents aged 12–13 years reported longer mean sleep duration than those aged 14–17 years (8.93 vs. 7.99 h per night; *p* < 0.0001) (Table 2). Male adolescents reported longer sleep than female adolescents (8.54 vs. 8.05 h per night; *p* = 0.0001). Mean sleep duration did not differ significantly across race/ethnicity categories (overall *p* = 0.10) or between adolescents from households below versus at or above 300% of the federal poverty level (8.39 vs. 8.21 h per night; *p* = 0.21).

### 2.3. Associations Between Sleep Measures and BMI

In the core model adjusted for age, sex, race/ethnicity, household poverty category, and survey year, weeknight sleep duration was not associated with BMI (β = −0.371 kg/m^2^ per additional hour; SE = 0.263; 95% CI: −0.890–0.149; *p* = 0.161; Table 3). A diet-adjusted sensitivity model that additionally included previous-day fruit and vegetable servings and any consumption of sweetened fruit drinks, sports drinks, or energy drinks produced a similar estimate (β = −0.366; SE = 0.267; 95% CI: −0.894–0.162; *p* = 0.173). The estimate also remained similar in the broader diet- and behavior-adjusted model (β = −0.359; SE = 0.268; 95% CI, −0.889–0.171; *p* = 0.183).

The sleep-duration-by-age-group interaction was statistically significant in the core model (*p* for interaction = 0.042). Sleep duration was not associated with BMI among adolescents aged 12–13 years (β = 0.134; SE = 0.428; 95% CI: −0.711–0.979; *p* = 0.755), whereas each additional hour of sleep was associated with a 0.895-kg/m^2^ lower BMI among adolescents aged 14–17 years (β = −0.895; SE = 0.288; 95% CI: −1.464–−0.326; *p* = 0.002). After additional dietary and behavioral adjustment, the interaction was attenuated (*p* for interaction = 0.050). However, a sensitivity analysis treating age as a continuous variable did not identify effect modification (*p* for interaction = 0.36). Sleep-duration interactions by sex and race/ethnicity were not statistically significant.

After adjustment for sleep duration and core covariates, bedtime (β = 0.282 kg/m^2^ per 1 h later; SE = 0.319; *p* = 0.379), wake time (β = 0.299; SE = 0.336; *p* = 0.376), and sleep midpoint (β = 0.293; SE = 0.330; *p* = 0.376) were not independently associated with BMI. The estimates remained nonsignificant after additional dietary and behavioral adjustment. An exploratory bedtime-by-age-group interaction was observed (*p* for interaction = 0.029), although neither age-specific bedtime slope was statistically significant. The wake-time-by-age-group interaction was not statistically significant (*p* for interaction = 0.379), nor was the sleep-midpoint-by-age-group interaction (*p* for interaction = 0.066). No sleep-timing interactions by sex or race/ethnicity were evident.

The overall sleep-duration estimate was also stable in analyses restricted to adolescents who attended school during the preceding week, those reporting 4–12 h of sleep, and those in the active-commuting domain (estimates not shown). There was no evidence of a quadratic association between sleep duration and BMI (*p* = 0.83) or heterogeneity by survey year (*p* for interaction = 0.29; models not shown). In a 2017-only sensitivity analysis that additionally adjusted for regular soda consumption, sleep duration was not significantly associated with BMI (β = 0.064 kg/m^2^ per additional hour; SE = 0.455; 95% CI, −0.842–0.970; *p* = 0.889; n = 448).

### 2.4. Exploratory Analysis of Sleep Duration and Overweight or Obesity

Sleep duration was not associated with the odds of having overweight or obesity in either the core model (OR = 0.89 per additional hour; 95% CI, 0.68–1.15; *p* = 0.36) or the model additionally adjusted for dietary and behavioral factors (OR = 0.88; 95% CI, 0.68–1.15; *p* = 0.36) (Table 4). There was no evidence that this association differed by age group (*p* = 0.18). In age-stratified analyses, sleep duration was not associated with weight-status classification among adolescents aged 12–13 years (OR = 1.13, 95% CI: 0.67–1.92; *p* = 0.64). Among adolescents aged 14–17 years, each additional hour of sleep was associated with lower odds of being classified as having overweight or obesity, although these results were only marginally significant (OR = 0.75, 95% CI: 0.57–1.00; *p* = 0.05). There was also no evidence of variation by sex (*p* for interaction = 0.96).

In exploratory analyses, evidence of variation by race/ethnicity was observed in the core model (global *p* for interaction = 0.04). The estimated sleep duration associations were OR = 1.06 (95% CI: 0.72–1.56; *p* = 0.76) among non-Latino White adolescents, OR = 0.59 (95% CI: 0.39–0.89; *p* = 0.01) among Latino adolescents, and OR = 1.16 (95% CI: 0.72–1.88; *p* = 0.53) among adolescents identifying with other or multiple racial groups. After additional adjustment for dietary and behavioral factors, the inverse association among Latino adolescents remained statistically significant (OR = 0.59, 95% CI: 0.39–0.90; *p* = 0.02), whereas the interaction was attenuated (global *p* for interaction = 0.05). There was no evidence that the overall association differed by survey year (*p* for interaction = 0.44).

## 3. Discussion

In this statewide representative sample of California adolescents, weeknight sleep duration was not associated with BMI or overweight/obesity overall, and the estimates were unchanged after adjustment for the available dietary and behavioral variables. In prespecified age group models, longer sleep was associated with lower BMI among older adolescents aged 14–17 years, but not among those aged 12–13 years. However, evidence of variation by age was not observed when age was modeled continuously. Sleep-timing measures were not independently associated with BMI after adjustment for sleep duration. Exploratory logistic models suggested possible race/ethnicity heterogeneity, with an inverse association among Latino adolescents, although the global interaction was attenuated after behavioral adjustment. Descriptively, adolescents aged 12–13 years reported nearly one additional hour of weeknight sleep compared with those aged 14–17 years, and male adolescents reported approximately half an hour more sleep than female adolescents. These differences should not be interpreted causally.

The absence of an overall association between sleep duration and BMI should be interpreted in the context of the study design and available measures. Sleep patterns and sleep opportunities change throughout adolescence due to developmental shifts in circadian timing and increasing academic and social demands [7,8]. Our sleep duration measure was derived from one-time reports of school-night bedtime and school-day wake time and is therefore best interpreted as a proxy for school-night time in bed. It did not capture night-to-night variability, weekend recovery sleep, sleep-onset latency, nighttime wakefulness, sleep quality, or sleep continuity. Sleep duration was also not associated with BMI-defined weight status in the overall sample. Although categorizing BMI can obscure variation within categories and generally provides less information than modeling BMI continuously, findings across both outcomes provided no evidence of an overall association between sleep duration and body size in this sample. The cross-sectional design further limits the ability to establish the temporal ordering of sleep duration and BMI.

California’s demographic diversity, which is higher than that of the US national population [13], makes statewide analyses useful for examining variation in sleep–health associations. Prior studies have reported racial/ethnic differences in adolescent sleep and in associations between sleep and adiposity [1,9,17,18,19,20,21]. In the exploratory logistic models, longer sleep duration was associated with lower odds of being classified as having overweight or obesity among Latino adolescents, but not among non-Latino White adolescents or adolescents identifying with other or multiple racial groups. Evidence of variation by race/ethnicity was attenuated after adjustment for dietary and behavioral factors, indicating that the subgroup pattern was sensitive to model specification. Because the dietary and behavioral variables could represent confounders, mediators, or consequences of sleep and weight status, attenuation after adjustment should not be interpreted as evidence that these factors explain the observed race/ethnicity differences. Given the cross-sectional design, these findings should not be interpreted as evidence that longer sleep has a causal or uniformly protective association. The observed differences may reflect unmeasured structural, social, behavioral, or developmental factors and warrant confirmation in longitudinal studies designed to investigate these potential explanations.

The inverse sleep–BMI association among adolescents aged 14–17 years persisted after adjustment for the available dietary and behavioral variables. Older adolescents experience biologically driven circadian phase delay alongside increasing social and academic demands and early school schedules, which may restrict sleep opportunity [7,8]. A prospective cohort of adolescents aged 14–18 years similarly found that longer sleep duration was associated with lower BMI across multiple quantiles of the BMI distribution [5]. Nevertheless, effect modification was not detected when age was modeled continuously, suggesting that the grouped finding may reflect a nonlinear developmental pattern or sensitivity to the selected 12–13-year versus 14–17-year threshold. Although older adolescents also had lower estimated odds of having overweight or obesity with each additional hour of sleep, the sleep-duration-by-age-group interaction was not statistically significant for this outcome. Thus, evidence of age-related variation was limited to continuous BMI and depended on both the outcome definition and how age was modeled. Replication in larger longitudinal samples with pubertal-stage measures is needed to determine whether the observed age-related differences are robust and reflect developmental changes associated with puberty rather than the selected age group cutoff.

We found no evidence that the association of sleep duration with BMI or overweight/obesity differed by sex. Prior studies have reported mixed sex-specific findings [22,23], which may reflect variability in study contexts by age, developmental stage, sleep measurement, outcome definition, and social factors. In our data, the sleep duration estimates were similar and not significant between female and male adolescents in both the continuous-BMI and overweight/obesity models.

Bedtime, wake time, and sleep midpoint were not independently associated with BMI after adjustment for sleep duration. Evidence regarding sleep timing and pediatric adiposity remains mixed, and the relevance of timing may depend on how circadian misalignment is operationalized [7]. Measures such as weekend–weekday variability, chronotype, and social jetlag may better capture misalignment than a single school-night schedule. An exploratory bedtime-by-age group interaction was observed, but neither age-specific slope was statistically significant. Given the number of interaction tests and the absence of weekend timing and chronotype measures, this finding may reflect chance variation and should not be emphasized as confirmatory evidence.

### Strengths and Limitations

Strengths of this study include the state-representative CHIS sample, calibrated person-level survey weights, and jackknife replicate-weight variance estimation. The analyses accounted for survey year and incorporated the available dietary and behavioral covariates collected in both survey cycles. Overall conclusions were similar across the primary models and sensitivity analyses restricted by recent school attendance and a prespecified sleep range, as well as analyses conducted in the active-commuting domain, examining variation by survey year and a potential nonlinear association, and, for 2017, additionally adjusting for regular soda consumption.

Several limitations should be acknowledged. The cross-sectional design precludes establishing temporality or causality. The sleep measure was derived from one-time reports of school-night bedtime and school-day wake time and is best interpreted as a proxy for school-night time in bed. It did not account for sleep-onset latency, nighttime wakefulness, or night-to-night variability. Height and weight were also self-reported. Furthermore, the use of BMI rather than BMI-for-age z-scores may be particularly relevant to comparisons across this broad age range because BMI changes with adolescent growth and maturation. The public-use files did not include exact age in months, BMI-for-age z-scores, pubertal stage, chronotype, weekend sleep timing, social jetlag, ambient or device-related light exposure, actual screen duration, 24-h activity patterns, comprehensive or objectively measured physical activity, or detailed dietary composition. The available dietary variables were limited to selected previous-day intake measures and may not reflect usual dietary intake. Sedentary time, active commuting, park activity, and household electronics rules were imperfect proxies for relevant behavioral characteristics. Consequently, residual confounding by developmental maturation, dietary intake, physical activity, and other unmeasured factors cannot be excluded.

The released CHIS variables contained no missing values following item-level imputation, although uncertainty related to imputation and measurement error remains. The sample size also limited precision in subgroup analyses, particularly for interaction tests. In addition, the available race/ethnicity categories combined adolescents from heterogeneous backgrounds within broad groups, including the other or multiple races category. These classifications may obscure differences in social context, discrimination, immigration experiences, and other structural factors relevant to sleep and BMI. Finally, multiple interaction tests were conducted, increasing the possibility of chance subgroup findings. Although the survey estimates are representative of California adolescents included in CHIS, the findings may not generalize to adolescents in other states, countries, or populations with different demographic, educational, and social contexts.

## 4. Materials and Methods

### 4.1. Design and Sample

We conducted a cross-sectional analysis of the 2017–2018 California Health Interview Survey (CHIS) adolescent data. CHIS is a population-based telephone survey designed to produce representative estimates of California’s noninstitutionalized population using a dual-frame random-digit-dial sampling design with multistage geographic stratification. Adolescents aged 12–17 years were interviewed directly after parental consent was obtained. Data for the 2017–2018 CHIS cycle were collected between June 2017 and January 2019. The 2017–2018 cycles were used because they represent the most recent CHIS waves that included detailed adolescent sleep measures; these items were discontinued in subsequent survey cycles.

No a priori sample size calculation was conducted because this study was a secondary analysis of an existing population survey, and all eligible respondents were included. The pooled public-use files contained 880 adolescents aged 12–17 years (n = 448 from 2017 and n = 432 from 2018) representing approximately 3.14 million California adolescents. Eligibility criteria were age 12–17 years and availability of a valid pooled full-sample survey weight. Because CHIS performs item-level imputation, the released variables used in the principal models had no missing values; therefore, no eligible respondents were excluded, and all 880 were included in the primary analyses. Sensitivity analyses included 684 adolescents who had attended school during the preceding week and 877 adolescents who reported 4–12 h of sleep on weeknights. Analyses were conducted from February through July 2026.

This study was a secondary analysis of publicly available, de-identified California Health Interview Survey Public-Use Files. The authors had no interaction with survey participants and had no access to direct identifiers or information through which participants’ identities could readily be ascertained. Accordingly, the present analysis did not constitute human-subjects research requiring Institutional Review Board review under 45 CFR 46.102(e)(1). The study was conducted in accordance with applicable ethical standards and the principles of the Declaration of Helsinki. For the original CHIS data collection, adolescents were interviewed directly following parental permission, and participation was voluntary.

### 4.2. Measures

Weeknight sleep duration was obtained from the CHIS-derived variable SLPH, based on adolescents’ reported school-night bedtime and school-day wake time. We independently calculated the bedtime-to-wake interval using modular arithmetic and found exact agreement with SLPH for 879 of 880 respondents. Because this interval does not account for sleep-onset latency or wakefulness during the night, it is best interpreted as a proxy for school-night sleep duration or time in bed. Bedtime was recorded in minutes after midnight; values after midnight were shifted forward by 24 h to create a continuous evening-to-morning scale. Wake time was expressed in hours after midnight. Sleep midpoint was calculated as bedtime plus one-half of the bedtime-to-wake interval and linearized around midnight, with pre-midnight values represented as negative hours and post-midnight values as positive hours. Timing variables were modeled per one-hour later timing.

Body mass index (BMI; kg/m^2^) was derived from self-reported height and weight using the CHIS public-use recode and was analyzed as a continuous outcome. The public-use files do not provide BMI-for-age z-scores or exact age in months, which is required to calculate valid CDC BMI z-scores. Therefore, a z-score outcome could not be derived without introducing age-classification error. CHIS-provided age- and sex-specific weight-status categories (underweight, normal weight, overweight, and obesity) were reported descriptively.

For secondary exploratory analyses, overweight or obesity was defined using the CHIS variable OVRWT2, which classifies adolescents with BMI at or above the 85th percentile for age and sex as having overweight or obesity. This binary outcome was examined using survey-weighted logistic regression.

Age was analyzed continuously and additionally categorized as 12–13 versus 14–17 years for prespecified interaction analyses. Sex was recorded in CHIS as male or female. Race/ethnicity was represented using the CHIS-provided combined recode and categorized as Latino, non-Latino White, or other or multiple races. Importantly, race/ethnicity was conceptualized as a social construct that may reflect differences in social and structural conditions rather than innate biological characteristics. Household poverty level was categorized as less than 300% versus 300% or more of the federal poverty level. The survey year was included in all pooled models. Sensitivity models additionally included previous-day fruit and vegetable intake, consumption of any sweetened fruit, sports, or energy drinks, weekday sedentary time, activity during the most recent park visit, and household rules for putting away electronics. Active commuting from school was evaluated in a domain analysis because the relevant questions were not applicable to all respondents. A 2017-only model additionally adjusted for regular soda consumption.

### 4.3. Statistical Analysis

CHIS performs item-level imputation, and sample-flow checks were used to assess the completeness of the released analytic variables separately for the core, sleep-timing, and extended models. These checks indicated that the variables used in the primary models had no missing values. The primary analyses, therefore, included the full eligible sample, and no respondents were excluded because of item nonresponse. Restricted-sample sensitivity analyses were conducted as survey-domain analyses to preserve appropriate design-based variance estimation.

Weighted means, percentages, standard errors, and 95% confidence intervals were estimated using pooled person-level sampling weights. Each annual full-sample weight was divided by two when the 2017 and 2018 survey cycles were pooled. Variance estimation incorporated 160 pooled jackknife replicate weights to account for the complex survey design. Survey year was included in all pooled BMI and overweight-or-obesity regression models. Descriptive differences in sleep duration by age group, sex, race/ethnicity, and household poverty category were evaluated using survey-weighted linear regression.

Survey-weighted linear regression was used to estimate associations of sleep duration, bedtime, wake time, and sleep midpoint with continuous BMI. Core models adjusted for age, sex, race/ethnicity, household poverty category (<300% versus ≥300% of the federal poverty level), and survey year. To assess whether timing was associated with BMI independently of sleep duration, bedtime, wake time, and midpoint models additionally adjusted for sleep duration. To assess potential confounding by dietary intakes, a diet-specific sensitivity model additionally included previous-day fruit intake servings, vegetable intake servings, and any sweetened fruit, sports, or energy drink consumption. A broader diet and behavior sensitivity model included both the dietary variables and weekday sedentary time, activity during the most recent park visit, and household rules for putting away electronics.

Because data on regular soda consumption were available only in 2017, an additional sensitivity analysis was restricted to 2017 respondents and adjusted for any regular soda consumption. This analysis used the original 2017 full-sample weight and 80 annual jackknife replicate weights. The results of this model were reported narratively because it was restricted to a single survey year.

For continuous BMI, effect modification was evaluated in separate core models using sleep-duration-by-age-group, sleep-duration-by-sex, and sleep-duration-by-race/ethnicity interaction terms. The age-group interaction was also evaluated after adjustment for dietary and behavioral factors. Subgroup-specific slopes and 95% confidence intervals were estimated from each interaction model, and the sleep-duration-by-race/ethnicity interaction for continuous BMI was evaluated using a joint two-degree-of-freedom test. Interactions of bedtime, wake time, and sleep midpoint with age group, sex, and race/ethnicity were also examined in models adjusted for sleep duration.

Additional BMI sensitivity analyses examined the sleep-duration-by-continuous-age interaction, the sleep-duration-by-survey-year interaction, a quadratic sleep-duration term, restriction to adolescents who attended school during the preceding week, restriction to adolescents reporting 4–12 h of sleep, and analysis within the applicable active-commuting domain. Survey-weighted logistic regression was used to estimate the odds of having overweight or obesity per additional hour of sleep. Both the core and extended sensitivity models used the same respective covariate sets as the sleep-duration linear models. Core interaction models examined whether the association varied by age group, sex, race/ethnicity, and survey year. The age-group and race/ethnicity interaction models were also evaluated after adjustment for dietary and behavioral factors. Subgroup-specific odds ratios and 95% confidence intervals were calculated from the fitted coefficients. Race/ethnicity interactions were evaluated using joint two-degree-of-freedom tests. Because the overweight-or-obesity and interaction analyses were exploratory and multiple interaction tests were conducted, the subgroup findings were interpreted with caution.

All statistical tests were two-sided, and *p* < 0.05 was considered statistically significant. All analyses were conducted using SAS version 9.4 (SAS Institute Inc., Cary, NC, USA).

## 5. Conclusions

In this state-representative sample of California adolescents, reported weeknight sleep duration was not associated with BMI or BMI-defined weight status overall, and findings were similar after adjustment for available dietary and behavioral factors. In prespecified age group analyses, longer sleep duration was associated with lower BMI among adolescents aged 14–17 years; however, evidence of variation by age was not observed when age was modeled continuously or when BMI-defined weight status was examined. Sleep-timing measures were not independently associated with BMI. Exploratory analyses suggested possible variation by race/ethnicity in the association between sleep duration and BMI-defined weight status, but this evidence was attenuated after additional adjustment. Longitudinal studies incorporating objective and repeated sleep measures, measured height and weight, and comprehensive assessments of circadian, developmental, dietary, and activity-related factors are warranted.

## Figures and Tables

**Table 1:** Characteristics of adolescents aged 12–17 years (CHIS 2017–2018) [14].

Participant Characteristic	Weighted Mean (SE) or n (%)
Age, years	14.51 (0.05)
Sex	
Male	457 (51.12)
Female	423 (48.88)
Race/ethnicity	
Latino	164 (32.35)
Non-Latino White	431 (28.75)
Other or multiple races	285 (38.90)
Body mass index, kg/m^2^	22.55 (0.27)
Weight status	
Underweight	39 (2.87)
Normal weight	574 (64.55)
Overweight	148 (15.47)
Obesity	119 (17.10)
Overweight or obesity	267 (32.57)
Poverty level category	
0–99% FPL	113 (17.88)
100–199% FPL	153 (21.54)
200–299% FPL	92 (10.50)
300% FPL and above	522 (50.09)
Weeknight sleep duration, hours	8.30 (0.07)
Sleep midpoint, hours after midnight	2.18 (0.06)
Wake time, hours after midnight	6.33 (0.06)
Bedtime, linearized hours	22.03 (0.07)
Fruit intake servings (previous day)	1.95 (0.09)
Vegetable intake servings (previous day)	1.40 (0.08)
Weekday sedentary time, hours	3.26 (0.21)
Weekend sedentary time, hours	4.80 (0.21)

Abbreviations: BMI, body mass index; FPL, federal poverty level. Bedtime was linearized by treating after-midnight values as occurring after 24:00. Sleep midpoint was expressed as signed hours relative to midnight. Values are weighted means (SE) for continuous variables and unweighted n (weighted %) for categorical variables. Dietary variables reflect intakes in servings on the previous day. Percentages may not total 100% due to rounding.

**Table 2:** Weeknight sleep duration by participant characteristics.

Participant Characteristic	N	Sleep Duration, Hours per Night Weighted Mean (SE)	*p*
Age group			
12–13 years	297	8.93 (0.10)	<0.0001
14–17 years	583	7.99 (0.09)	
Sex			
Male	457	8.54 (0.09)	0.0001
Female	423	8.05 (0.09)	
Race/ethnicity			
Latino	164	8.50 (0.14)	0.10
Non-Latino White	431	8.32 (0.11)	
Other or multiple races	285	8.13 (0.11)	
Poverty level category			
<300% FPL	358	8.39 (0.10)	0.21
≥300% FPL	522	8.21 (0.09)	

Abbreviation: FPL, federal poverty level. *p*-values are estimated using survey-weighted linear regressions. The poverty comparison used the prespecified <300% versus ≥300% FPL categorization.

**Table 3:** Primary and selected sensitivity analyses of associations of sleep duration and timing with body mass index.

Exposure Model	β (SE)	*p*	*p* for Interaction
Weeknight sleep duration, per additional hour			
Overall core model	−0.371 (0.263)	0.161	
Overall diet-adjusted model (sensitivity)	−0.366 (0.267)	0.173	
Overall diet- and behavior-adjusted model (sensitivity)	−0.359 (0.268)	0.183	
Age group, core model			0.042
Ages 12–13 years	0.134 (0.428)	0.755	
Ages 14–17 years	−0.895 (0.288)	0.002	
Age group, diet- and behavior-adjusted model (sensitivity)			0.050
Ages 12–13 years	0.180 (0.447)	0.688	
Ages 14–17 years	−0.861 (0.299)	0.005	
Sleep timing, per 1 h later			
Bedtime, adjusted for sleep duration	0.282 (0.319)	0.379	
Bedtime, adjusted for sleep duration, diet, and behavior	0.269 (0.334)	0.421	
Wake time, adjusted for sleep duration	0.299 (0.336)	0.376	
Wake time, adjusted for sleep duration, diet, and behavior	0.288 (0.352)	0.415	
Sleep midpoint, adjusted for sleep duration	0.293 (0.330)	0.376	
Sleep midpoint, adjusted for sleep duration, diet, and behavior	0.281 (0.345)	0.417	
Sleep-timing interactions by age group			
Bedtime × age group			0.029
Ages 12–13 years	−0.585 (0.605)	0.335	
Ages 14–17 years	0.677 (0.386)	0.081	
Wake time × age group			0.379
Ages 12–13 years	0.020 (0.632)	0.975	
Ages 14–17 years	0.634 (0.401)	0.116	
Sleep midpoint × age group			0.066
Ages 12–13 years	−0.788 (0.769)	0.307	
Ages 14–17 years	0.775 (0.430)	0.073	

Note. Table 3 presents the primary models and selected sensitivity and interaction analyses. Additional sensitivity and interaction results are reported in the text but are not tabulated. β coefficients represent the difference in BMI (kg/m^2^) associated with each additional hour of weeknight sleep or each 1-h-later sleep-timing measure. Core models adjusted for age, sex, race/ethnicity, household poverty category, and survey year. The diet-adjusted model additionally included previous-day fruit and vegetable servings and any consumption of sweetened fruit drinks, sports drinks, or energy drinks. Diet- and behavior-adjusted models additionally included weekday sedentary time, activity during the most recent park visit, and household electronics rules. Sleep-timing models additionally adjusted for sleep duration. Age-specific estimates from the interaction models represent simple slopes within each age group. *p*-values for interaction were obtained from design-adjusted Wald tests of the exposure-by-age-group product terms.

**Table 4:** Sleep duration and odds of overweight or obesity.

Model or Stratum	OR (95% CI) per Additional Hour of Sleep	*p*	*p* for Interaction
Overall association			
Core model	0.89 (0.68–1.15)	0.36	
Sensitivity-adjusted model	0.88 (0.68–1.15)	0.36	
Age group, core model			0.18
12–13 years	1.13 (0.67–1.92)	0.64	
14–17 years	0.75 (0.57–1.00)	0.05	
Age group, sensitivity-adjusted model			0.18
12–13 years	1.14 (0.66–1.95)	0.64	
14–17 years	0.75 (0.56–1.00)	0.05	
Sex, core model			0.96
Female	0.88 (0.62–1.25)	0.48	
Male	0.89 (0.64–1.23)	0.48	
Race/ethnicity, core model			0.04
Non-Latino White	1.06 (0.72–1.56)	0.76	
Latino	0.59 (0.39–0.89)	0.01	
Other or multiple races	1.16 (0.72–1.88)	0.53	
Race/ethnicity, sensitivity-adjusted model			0.05
Non-Latino White	1.08 (0.73–1.61)	0.69	
Latino	0.59 (0.39–0.90)	0.02	
Other or multiple races	1.16 (0.72–1.88)	0.53	
Survey year			0.44
2017	0.98 (0.65–1.47)	0.92	
2018	0.81 (0.60–1.09)	0.17	

Odds ratios represent the odds of having overweight or obesity associated with each additional hour of weeknight sleep. Core models adjusted for age, sex, race/ethnicity, household poverty category, and survey year. Sensitivity-adjusted models additionally included previous-day fruit intake servings, vegetable intake servings, any sweetened fruit/sports/energy drink consumption, weekday sedentary time, activity during the most recent park visit, and household electronics rules. *p*-values for interaction were obtained from design-adjusted Wald tests. The race/ethnicity interaction was evaluated using a 2-degree-of-freedom global test; age group, sex, and survey-year interactions were evaluated using 1-degree-of-freedom tests.

## Data Availability

The data used in this study are publicly available from the California Health Interview Survey through the UCLA Center for Health Policy Research (https://healthpolicy.ucla.edu/chis). The data were accessed on 3 March 2026. Researchers may obtain access to these data directly from the data provider.
